# The Medical Corps of the United States Navy

**Published:** 1888-03

**Authors:** 


### THE MEDICAL CORPS OF THE UNITED STATES NAVY.

In the *Reference Handbook of the Medical Sciences*, Medical-Director A. L. Gihon, of the United States Navy, says:

Admission into the medical corps of the navy can only be obtained through a professional examination before the naval medical board, composed of officers of the higher grades. From the beginning this board has assumed to be the sole judge of the qualifications of candidates, the mere possession of the diploma of a medical school not being recognized as even presumptive evidence of professional abilities and acquirements. Inquiry is first made as to whether the candidate has received a liberal education, which is properly considered an indispensable pre-requisite to an intelligent comprehension of the science of medicine. An autobiographical sketch, an essay on some assigned professional topic, and written answers to a series of comprehensive questions in the various departments of medicine, are intended as evidences of such preliminary proficiency, the orthography and punctuation, grammatical construction, form, and manner of expression being as carefully scrutinized as accuracy of statement. An oral examination follows by the several members of the board, in the presence of all, upon every branch of medicine and upon such collateral studies as the candidate may have pursued, with the object, not merely of ascertaining the amount of detailed information he may have acquired by rote, but rather the extent of his intelligent understanding of the fundamental facts and principles which constitute the science of medicine. Finally, extemporaneous chemical and pharmaceutic exercises, the clinical diagnosis and treatment of actual patients in hospital, the adjustment of surgical apparatus and appliances, and the performance of operations upon the cadaver, exhibit his acquaintance with the practical requirements of the healing art, and his fitness to assume its responsibilities under the emergencies which sometimes place the issue of life or death upon his unaided knowledge and skill.

Manifestly, such a course of examination, written, oral, and practical, adequately determines whether the candidate has received a proper education in medicine, and the examination is only such as should be exacted of every aspirant for a doctor's degree, and such as can be hoped for only when boards of examiners shall be instituted entirely independent of teaching faculties. A second examination, after a lapse of three (formerly five) years, is required for passing out of the grade of assistant surgeon preliminary to promotion to that of surgeon. This examination presupposes a wider practical acquaintance with the various branches of medicine and a familiarity with its current literature. Similarly, in civil life, it would be well if the graduate should first receive only a baccalaureate degree (M. B.), and as an incentive to application and study have the M. D. before him as the reward of a second examination five years later. But the interest of the bureau of medicine and surgery in the young naval medical officer does not cease with this final examination. Opportunities for self-improvement are liberally provided, and individual research and investigation are encouraged by the supply of apparatus, instruments, or other facilities required, and by the publication of essays voluntarily contributed, as well as by the requirement of annual statistical, medical, and sanitary reports from every officer in charge of the medical department of a vessel or station, embracing the medical topography, climatology, and hygiene of every station or place visited, with all attainable information respecting statistics of disease and its causes, establishments for the care of the sick, charitable institutions, medical colleges, and other matters of professional interest. Again, in civil life, this might be paralleled by the more general employment of young men as salaried attachés of hospitals, dispensaries, asylums, infirmaries, cliniques, and other eleemosynary medical institutions, where, under the supervision of older men, that experience might be acquired which alone justifies any one in accepting the responsibility of human lives, and where similar provisions for professional research might be afforded, which are not possible in a young graduate's meagrely-appointed office.

The medical officer of the navy having passed the rigorous examination for admission, is made to understand at the outset of his career that his first duty, as a physician, is to protect the community in which he is established from the ravages of disease; and herein the medical corps of the navy merely exemplifies the real mission of the medical man in every walk in life—to place those who are under his care in the best possible sanitary condition, to jealously guard against the operation of preventable causes of disease, to antagonize the morbific agencies that elude their vigilance, or find access by inscrutable ways, and thus to reduce to a minimum the necessity for therapeutic measures; and finally to use natural agencies for purposes of cure wherever these can replace pharmaceutic administration.

The sanitary supervision of a community like the navy is much simplified by the preliminary physical examination of all the men and boys—adults and adolescents—who enter it, and by their compulsory subjection to regulated habits in food, drink, personal cleanliness, etc. The immature and the very old, the weak and decrepit, women with their special train of ailments, and those predisposed to disease—classes which in civil life outnumber all the rest—are excluded from enlistment. Still, it is within the province, as it is the duty, of the civil practitioner, to inculcate, far more strongly than he does now, hygienic principles, and to enforce restraints, as in preventing the marriage of individuals with transmissible taints.

